# Locally Advanced Rectal Cancer: Does Assessment of Response After Neoadjuvant Chemoradiotherapy Impact Management?

**DOI:** 10.6004/jadpro.2013.4.6.9

**Published:** 2013-11-01

**Authors:** Elizabeth A. Wolf, Daniel L. Malatek

**Affiliations:** From MD Anderson Cancer Center, Houston, Texas

Colorectal cancer, the fourth most common malignancy worldwide, accounts for 12% of all cancer deaths in the United States. According to National Cancer Institute (NCI) Surveillance, Epidemiology, and End Results (SEER) data estimates, 142,820 men and women will be diagnosed with colorectal cancer in 2013 (NCI, 2013). Rectal cancer is a subset of lower gastrointestinal (GI) malignancies and occurs with relatively common frequency. By definition, rectal malignancies begin anatomically just above the anal sphincter complex and extend proximally 12 to 15 cm (Fry, Mahmoud, Maron, & Bleier, 2008).

The main histology of most rectal cancers is adenocarcinoma, which will be the subject of this article. In contrast to colon malignancies, the treatment for locally advanced rectal cancer typically involves some form of neoadjuvant chemoradiotherapy. For some locally advanced tumors, patients may achieve a complete pathologic response following neoadjuvant treatment. The decision to avoid radical surgery for those with complete pathologic response continues to be a provocative debate. To understand this debate, one has to consider the historical background and how standard treatment for locally advanced rectal cancer came into use.

## Background

Prior to the mid-1980s, patients diagnosed with rectal cancer usually underwent surgery alone. At that time, surgery was not standardized to total mesorectal excision (TME), and chemoradiotherapy was not routinely offered prior to surgery. The results often led to high rates of pelvic failure. Numerous randomized clinical trials investigating the adjuvant use of chemotherapy and radiation therapy to improve outcomes were performed from the 1980s through the 1990s (Czito & Willett, 2008). The findings of these trials resulted in the 1990 National Institutes of Health (NIH) Consensus Conference statement that supported postoperative chemoradiotherapy as the standard of care for stages II and III rectal cancers, based mostly on the results of three randomized trials: the Gastrointestinal Study Group (GITSG) trial, the North Central Cancer Treatment Group (NCCTG) trial, and the National Surgical Adjuvant Breast and Bowel Project (NSABP) R01 trial (Czito & Willett, 2008; NIH, 1990).

Then, in 1997, published results of the Swedish Rectal Cancer Trial (SRCT) reported that preoperative radiotherapy followed by curative surgery reduced the rates of local recurrence and improved survival among patients with resectable rectal cancer (SRCT, 1997). In addition, a long-term follow-up analysis of this trial has further shown that node-positive patients who undergo preoperative radiotherapy with TME have a lower rate of local recurrence (Folkesson et al., 2005).

Another significant trial supporting the use of preoperative chemoradiotherapy was the 2001 so-called "Dutch Trial," which compared TME alone to preoperative chemoradiotherapy followed by TME, with findings of improvement in local failure rates for stages II and III in the preoperative arm than in the surgery alone arm (Czito & Willett, 2008).

More recently, the German Rectal Cancer Study comparing preoperative chemoradiotherapy vs. postoperative therapy has shown that preoperative chemoradiotherapy results in superior pelvic control and increased sphincter preservation as well as lower rates of acute and chronic toxicity (Czito & Willett, 2008; Sauer et al., 2004). As such, preoperative chemoradiotherapy followed by curative surgery has been the recommended treatment for stages II and III rectal cancers, and even some select stage IV rectal cancers, from the late 1990s to the present. In considering this treatment, clinicians must assess the different variations of rectal tumors upon presentation.

## Clinical Presentation

**Figure 1:**
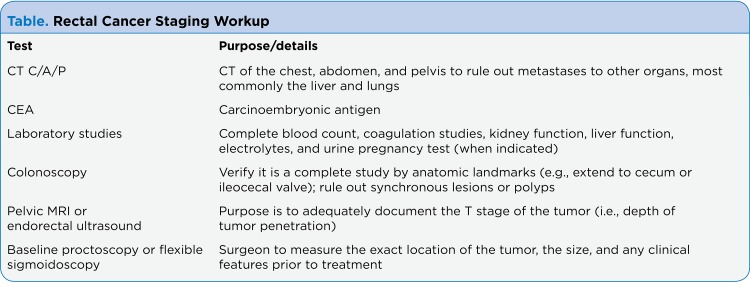
Figure 1. Colonoscopy revealed a rectal tumor with some ulceration and involvement of three-quarters of the wall of circumference.

Patients with rectal tumors typically present with symptoms such as rectal bleeding and/or rectal pain, with possible associated symptoms such as acute constipation or diarrhea episodes, fatigue, and weight loss. These patients should be properly evaluated with a thorough physical examination, including digital rectal exam (DRE). They should also be referred for a colonoscopy (Figure 1), with or without findings of a mass or lesion by DRE. Any suspicious masses, lesions, or polyps found on endoscopy should be biopsied for review by pathology.

When a patient presents with a pathologically confirmed rectal malignancy, a staging workup should be initiated (Table). Ideally, a colorectal specialist or GI surgeon should perform a physical exam with a DRE and proctoscopy. Radiographic imaging, including an endorectal ultrasound or pelvic MRI, should be obtained to assess pelvic tumor location, tumor status, and regional nodal staging. Consideration should be given to endoscopic marking or tattooing of the tumor due to the possibility of achieving a complete clinical response following treatment. However, oftentimes the scar formed from the tumor regression itself is used as the subsequent reference point for endoscopists and surgeons.

**Table 1:**
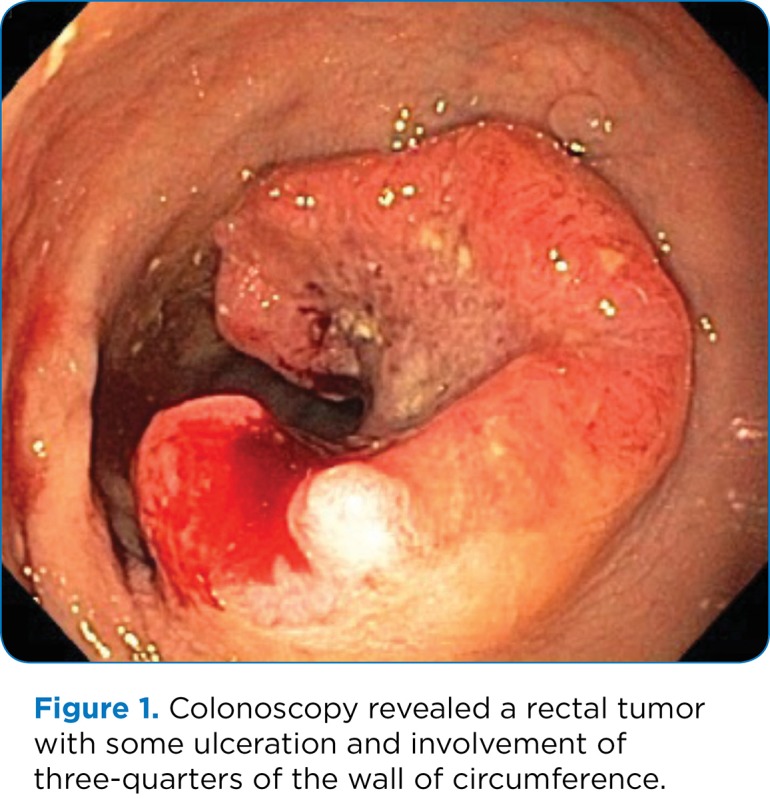
Table 1. Rectal Cancer Staging Workup

**Figure 2:**
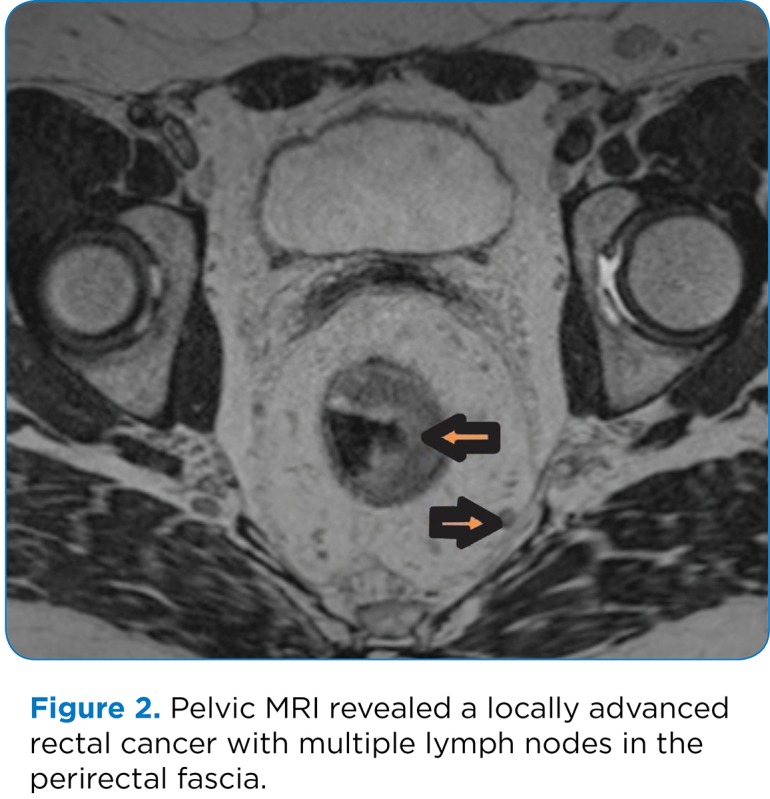
Figure 2. Pelvic MRI revealed a locally advanced rectal cancer with multiple lymph nodes in the perirectal fascia.

CT scans of the chest, abdomen, and pelvis are also obtained to determine the presence or absence of nodal and distant metastases. In some cases, the baseline CT will demonstrate pelvic node positivity, but it is not thought to be a substitute for pelvic staging by endoscopic ultrasound or MRI (Figure 2). Baseline bloodwork should include a complete blood count (CBC) with differential and a complete basic metabolic panel, including liver function tests, blood urea nitrogen, creatinine, electrolytes, and carcinoembryonic antigen levels. A urine pregnancy test is also indicated for women between the ages of 11 and 55 as well as for those who are still having menstrual cycles or had their last cycle within 6 months of diagnosis.

## Staging of Rectal Cancer and Role of Neoadjuvant Chemoradiotherapy

For patients with rectal tumors staged as T1 or T2, surgery alone is the recommended standard of care. For patients with locally advanced rectal tumors (T3, T4, or N+), a preoperative treatment regimen is recommended unless it is medically contraindicated. The recommended current standard of care is neoadjuvant concurrent chemoradiotherapy consisting of a total radiotherapy dose to the pelvis of 45 to 50 Gy delivered in 25 to 28 daily fractions followed by a tumor boost of 5.4 Gy in 3 daily fractions for preoperative radiation and a 5.4 to 9.0 Gy tumor bed boost in 3 to 5 daily fractions for postoperative radiotherapy with concurrent fluorouracil (either oral capecitabine or infusional 5-FU), followed by curative surgery approximately 6 to 8 weeks after completion of treatments (National Comprehensive Cancer Network [NCCN], 2013). Additionally, select patients are triaged to undergo treatment with a total radiotherapy dose to the rectum of 25 Gy delivered in 5 daily fractions followed by curative surgery approximately 1 week after completion of treatments (SRCT, 1997).

The goal of both of these treatment approaches is reduction of local recurrence rates. The longer course of 28 daily fractions may facilitate sphincter preservation for low-lying rectal tumors, as demonstrated by several clinical trials, such as the 1995 phase I/II trial performed by Bruce Minsky and colleagues at Memorial Sloan-Kettering Cancer Center (MSKCC) that showed an 83% conversion rate from an abdominoperineal resection (APR) to a low anterior resection (LAR) with coloanal anastomosis following longer course treatment (Minsky, Cohen, Enker, & Paty, 1995).

For the most part, these chemoradiotherapy approaches have shown that they are well-tolerated by patients, with side effects consisting mostly of mild abdominal cramping/diarrhea, mild nausea/vomiting, mild fatigue, mild dysuria, and a mild skin reaction that commonly subsides a few weeks after completion of treatment. Late side effects of radiotherapy, including almost certain infertility and other effects such as bowel urgency, proctitis, and sexual dysfunction, can affect up to 20% to 30% of patients. However, less than 10% of patients will suffer other late side effects of treatment such as chronic diarrhea, poor absorption of food, bladder and bowel complications, and insufficiency fractures.

The low risk of patients suffering debilitating acute and late side effects from treatments is considered acceptable in view of the benefits of therapy, including significant reduction in the risk of local recurrence and in low-lying distal rectal tumors and the possibility of conversion from an APR to sphincter-sparing surgery. Prior to initiation of any of these modalities for treatment, a discussion of the risks of each modality and the cumulative effects of treatment, both physically and on quality of life, should take place. Following completion of chemoradiotherapy treatments, an appropriate time interval of approximately 6 to 8 weeks is allowed for patients to recover from treatment side effects. During this period, attention is turned toward the surgical intervention and the process of assessing those patients prior to surgery.

## Preoperative Assessment

During the preoperative assessment, some GI/colorectal surgeons may elect to repeat imaging with CT scans of the abdomen and pelvis. Laboratory studies, including a CBC with differential and basic metabolic panel, should be obtained to verify that a patient’s liver and kidneys as well as other organ systems are functioning adequately. Appropriate cardiac evaluation is indicated for patients with known heart disease. A proctoscopy or flexible sigmoidoscopy is performed by the GI surgeon to elucidate the clinical response to treatment and to verify that no clinical evidence of progression of disease has occurred (to ensure that the surgical plan does not need to be amended). Reevaluation of the patient’s performance status following neoadjuvant treatment is also recommended to assess whether the patient is robust enough to withstand the rigors of surgery.

Preoperative counseling, an essential part of the preoperative workup, is an area that often involves both the advanced practitioner (AP) and the physician. Preoperative counseling includes revisiting the probable and possible impact of treatment on a patient’s quality of life, including potential issues beyond acute recovery. Both short- and long-term possible sequelae of surgery—including but not limited to bowel dysfunction, which presents in a wide range of issues; bowel frequency and urgency; bladder dysfunction; sexual dysfunction; fertility issues; and the possibility of a temporary or permanent stoma—should be discussed with the patient. The health-care team needs to revisit these issues with the patient in an ongoing fashion in the acute recovery phase as well as in the surveillance phase, as the side effects present themselves. Of note, quality-of-life issues have been found to be a significant factor in many decisions patients make regarding treatment; however, the topic of quality of life is beyond the current scope of this article.

## Types of Rectal Surgeries

The goal of cancer surgery is complete removal of a tumor with a margin of healthy tissue in all planes: superiorly, inferiorly, and radially. Adequate resection contributes to improvements in overall outcome, such as decreases in local recurrence and improvements in overall survival. For rectal malignancies, the type and extent of the surgery are mainly based on the location of the tumor, taking into consideration the patient’s performance status and other comorbidities. Specifically, the surgery proposed is not based on the T staging but rather on the anatomic location. Currently, all invasive GI malignancies are recommended to undergo TME; the name of the procedure—total mesorectal excision—reflects the amount and location of the tissue necessary to be removed to adequately treat the tumor.

**Figure 3:**
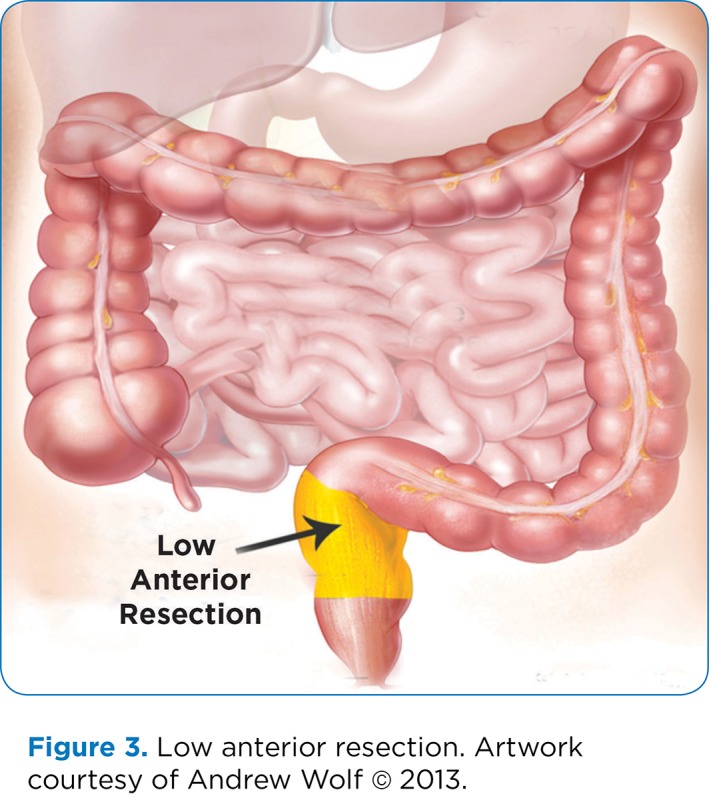
Figure 3. Low anterior resection. Artwork courtesy of Andrew Wolf © 2013.

**Figure 4:**
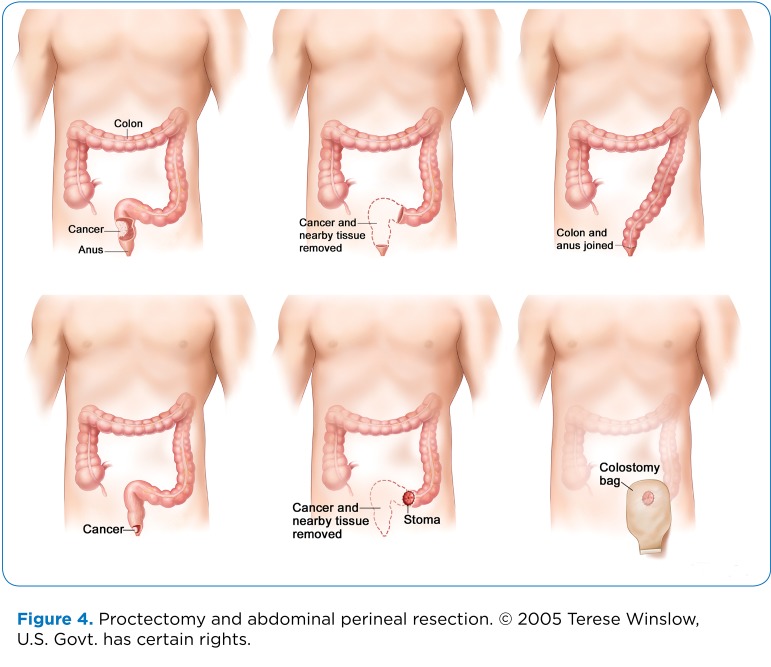
Figure 4. Proctectomy and abdominal perineal resection. © 2005 Terese Winslow, U.S. Govt. has certain rights.

The main types of rectal surgeries include the following:

- Low anterior resection, see Figure 3
- Proctectomy with coloanal anastomosis, see Figure 4
- Abdominal perineal resection, see Figure 4
- Pelvic exenteration (variable types)

For patients who need a pelvic exenteration, the exact operation in these cases is variable and dependent upon removal of the tumor and any involved adjacent pelvic organs, such as the bladder, prostate, or uterus. No one diagram exists to illustrate this surgery as it is tailored to the individual and the extent of tumor involvement.

Other procedures for sphincter preservation, such as transanal excision, do not garner lymph nodes and therefore do not involve a TME. Transanal excision is typically reserved for patients with stage 1 disease and other carefully selected cases, but it is not considered standard of care for patients with locally advanced rectal cancer. Less invasive surgical techniques may be beneficial as long as oncologic principles are properly followed, such that the tumor is removed completely with TME and a margin of healthy tissue. Organ preservation with less than total extirpation of the tumor and with an appropriate margin is not appropriate otherwise.

## Pathologic Response

Pathologic assessment of the patient’s surgical specimen provides useful information to help discern an individual’s response to neoadjuvant treatment as well as to clarify the role of additional therapy. Resected tumor specimens and all resected tissues are evaluated by a GI pathologist to determine final tumor and nodal surgical staging as well as features that have prognostic value: percentage of viable tumor cells, number of lymph nodes resected with or without metastatic involvement, and presence or absence of lymphovascular and perineural invasion.

Based on the pathologic findings, a patient’s response to treatments is reported as a complete response (CR), intermediate response, poor response, or other such nomenclature. Grading of tumor regression has also been used in the past to help characterize treatment stratification, but it is difficult to measure uniformly across multiple institutions and clinicians. Pathologic complete response (pCR) is the absence of tumor cells in the surgical specimen; predicting pCR based on clinical complete response (cCR) is a challenging concept (Minsky et al., 1995) that is important to mention but beyond the scope of this article.

The role of adjuvant therapy, including postoperative radiotherapy if not given preoperatively, is based on the surgical pathologic findings as defined by the NCCN guidelines (NCCN, 2013; see the barcode on page 439). Evaluating a patient’s pathologic response to neoadjuvant chemoradiotherapy may serve as an early prognostic indicator that is associated with long-term oncologic outcomes. It may also prove useful as a tool to guide individual therapy in patients, including possibly adopting a "wait-and-see" approach for select patients following neoadjuvant treatments with chemoradiotherapy in lieu of a curative surgery soon after completion of treatments. This approach has prompted an ongoing discussion in the literature, such as that seen in a recent systematic review by Glynne-Jones and Hughes (2012), who cite data from Habr-Gama and colleagues (2004) showing that select patients with cCR could benefit from a nonoperative approach with evidence of low local tumor recurrence rates and good overall survival. Habr-Gama’s provocative data have led to ongoing debate among clinicians.

Furthermore, a retrospective study from MSKCC looked at the outcomes of patients who had achieved cCR who were treated with "watchful waiting" compared with pCR patients who had undergone rectal resection (Smith et al., 2012). Although the study was small, it did suggest that for certain individuals, nonoperative management (NOM) might be a reasonable option to consider, since NOM appreared to achieve similar local and distant disease control when compared with salvage surgery. At present, though, to follow this wait-and-see approach, a thorough discussion between the provider and patient needs to occur. Patients need to make well-informed decisions balancing the potential risks and benefits of treatment. They also need to understand that the wait-and-see approach deviates from the current standard of care, and that there are no clear guidelines for measurement of cCR or the length of surveillance during observation.

As treatment strategies continue to improve for patients with locally advanced rectal cancer, focus is beginning to turn to other ways to improve patient outcomes and comorbidities associated with treatments. In addition to the possibility of adopting a wait-and-see approach for select patients with suspected cCR, some patients are considering other minimally invasive surgery options or possibly delaying surgery and receiving all chemotherapy in the neoadjuvant setting only. The possibility of using pathologic response to neoadjuvant treatments as a guideline for additional treatment modalities and for determining prognostic outcomes is also being investigated.

## MDACC Study

Findings of a large retrospective study by Park et al. that attempts to begin to address these questions, performed at the University of Texas MD Anderson Cancer Center (MDACC), were recently published in the Journal of Clinical Oncology (Park et al., 2012). The aim of this study was to compare the oncologic outcomes of patients with rectal cancer treated by preoperative chemoradiotherapy and radical resection stratified by degree of tumor response to chemoradiotherapy. Pretreatment clinical staging was compared to surgical pathologic staging, and patients were classified by tumor response: complete response vs. intermediate response vs. poor response. The 5-year recurrence-free survival, 5-year distant metastasis, and 5-year local recurrence rates were then compared with these three groups of tumor response.

This interesting and provocative study explores the response of locally advanced rectal patients to neoadjuvant therapy across 15 years at a single institution. It explores whether response to preoperative therapy can be used as an indicator of long-term outcomes. The findings question whether there are factors that could prompt new therapies, or other strategies to improve response to treatment in all tumors, in order to decrease recurrence rates and improve survival.

When compared to other recently published studies that evaluated pathologic tumor response to neoadjuvant therapy, the findings of Park and colleagues are quite similar. For example, a study published in December 2012 that retrospectively reviewed the outcomes of patients managed with selective NOM after a complete response to neoadjuvant treatment and compared those outcomes with patients who underwent standard rectal resection with a pCR. The findings were as follows: Among 265 patients treated neoadjuvant chemoradiotherapy and rectal resection, 57 patients (22%) had a pCR and formed the control group with a median follow-up of 43 months. Thirty-two patients were treated with NOM after a pCR with a median follow-up of 28 months. Six patients in the NOM group had local tumor recurrence with a median time of 11 months, and all were controlled by salvage rectal resection with no further local recurrence of disease. None of the rectal resection group patients suffered local failures. The 2-year distant disease-free survival rates were 88% vs. 98%, with an overall survival rate of 96% vs. 100% (Smith et al., 2012).

Another study published in the Journal of Clinical Oncology in October 2005 assessed the impact of tumor regression grading (TRG) and its value in correlation to established prognostic factors in a cohort of rectal cancer patients treated by preoperative chemoradiotherapy, reporting similar pathologic and survival findings. A designation of TRG4 was used to indicate patients with no viable tumor cells detected following preoperative CRT, TRG3 indicated regression of more than 50% with fibrosis outgrowing the tumor mass, TRG2 was defined as regression less than 50%, TRG1 was basically morphologically unaltered tumor, and the TRG0 group, indicated the complete absence of fibrosis. The 5-year disease-free survival rate after preoperative therapy and curative resection was 86% for the TRG4 group, 75% for the TRG2 and TRG3 groups, and 63% for the TRG0 and TRG1 groups (Rödel et al., 2005).

## Conclusion

Based on a series of data, including the recent MDACC study by Park and colleagues, treatment response to neoadjuvant chemoradiotherapy among patients with locally advanced rectal cancer undergoing radical resection can be an important early surrogate marker that correlates to oncologic outcomes. In addition, final pathologic stage is an early response indicator for long-term outcomes that provides better prognostication than does the clinical stage; patients who achieve a complete response and undergo radical resection have excellent prognosis with low risk for local or distant recurrence. Based on the treatment response stratification, the current NIH recommended surveillance guidelines for locally advanced rectal cancer should not be changed. These data provide guidance with response-stratified oncologic benchmarks for comparisons of novel treatment strategies.
